# Sequential decitabine and carboplatin treatment increases the DNA repair protein XPC, increases apoptosis and decreases proliferation in melanoma

**DOI:** 10.1186/s12885-018-4010-9

**Published:** 2018-01-26

**Authors:** Timothy Budden, Andre van der Westhuizen, Nikola A. Bowden

**Affiliations:** 1 Hunter Medical Research Institute and Faculty of Health, University of Newcastle, Newcastle, NSW Australia; 2 Department of Medical Oncology, Calvary Mater Hospital, Newcastle, NSW Australia

**Keywords:** Melanoma, Methylation, Decitabine, Carboplatin, XPC

## Abstract

**Background:**

Melanoma has two key features, an over-representation of UV-induced mutations and resistance to DNA damaging chemotherapy agents. Both of these features may result from dysfunction of the nucleotide excision repair pathway, in particular the DNA damage detection branch, global genome repair (GGR). The key GGR component XPC does not respond to DNA damage in melanoma, the cause of this lack of response has not been investigated. In this study, we investigated the role of methylation in reduced XPC in melanoma.

**Methods:**

To reduce methylation and induce DNA-damage, melanoma cell lines were treated with decitabine and carboplatin, individually and sequentially. Global DNA methylation levels, XPC mRNA and protein expression and methylation of the XPC promoter were examined. Apoptosis, cell proliferation and senescence were also quantified. XPC siRNA was used to determine that the responses seen were reliant on XPC induction.

**Results:**

Treatment with high-dose decitabine resulted in global demethylation, including the the shores of the XPC CpG island and significantly increased *XPC* mRNA expression. Lower, clinically relevant dose of decitabine also resulted in global demethylation including the CpG island shores and induced XPC in 50% of cell lines. Decitabine followed by DNA-damaging carboplatin treatment led to significantly higher XPC expression in 75% of melanoma cell lines tested. Combined sequential treatment also resulted in a greater apoptotic response in 75% of cell lines compared to carboplatin alone, and significantly slowed cell proliferation, with some melanoma cell lines going into senescence. Inhibiting the increased XPC using siRNA had a small but significant negative effect, indicating that XPC plays a partial role in the response to sequential decitabine and carboplatin.

**Conclusions:**

Demethylation using decitabine increased XPC and apoptosis after sequential carboplatin. These results confirm that sequential decitabine and carboplatin requires further investigation as a combination treatment for melanoma.

**Electronic supplementary material:**

The online version of this article (10.1186/s12885-018-4010-9) contains supplementary material, which is available to authorized users.

## Background

There are two features of melanoma that suggest a defect in DNA repair, an extremely high mutation load, indicative of unrepaired UV-induced DNA damage [1], and innate resistance to DNA damaging agents such as platinum based chemotherapies [2]. These can both be connected to the nucleotide excision repair (NER) pathway, the DNA repair system that is responsible for the removal of DNA damage that distorts the DNA helix, including UV photoproducts and platinum strand crosslinks [3].

The NER pathway consists of approximately 30 proteins that remove helix distorting lesions through for steps: a) damage recognition, b) unwinding of the DNA locally around damage, c) incision of damaged DNA by endonucleases, and d) DNA resynthesis and ligation [4]. There are two branches of damage recognition that lead to the common repair pathway: transcription coupled repair (TCR) and global genome repair (GGR). TCR is linked to active gene transcription and is initiated when RNA polymerase is stalled at DNA damage during transcription. GGR however is not dependent on transcription and scans the entire genome including both active and silent genes, and non-transcribed regions using DNA damage binding proteins XPC and UV-DDB (DDB1 and DDB2) [5].

Several previous studies have found an association between high or low levels of NER protein and mRNA levels and platinum chemoresistance (reviewed in [6]). The NER component ERCC1 has been extensively studied as a predictive biomarker for response to chemotherapy. To date, high levels of ERCC1 before platinum chemotherapy have been associated with poor response in melanoma [7], non-small cell lung cancer [8, 9], head and neck [10], gastric [11, 12], bladder [13] and oesophageal cancer [14]. Small molecule inhibitors of the ERCC1-XPF complex have been developed and shown to potentiate cisplatin efficacy in the A375 melanoma cell line [15] and H460 and H1299 lung cancer cell lines [16]. Further to this, in a melanoma mouse xenograft model loss of ERCC1 resulted in sensitivity to cisplatin [17]. To date, the study of NER in relation to platinum chemoresistance has largely focused on ERCC1.

In addition to the evidence supporting ERCC1 as a biomarker of platinum chemoresistance, our previous research has shown that the GGR damage detection branch of NER, does not function correctly in melanoma. We have found that the three GGR components XPC, DDB1 and DDB2 do not respond to UV treatment in melanoma cell lines, resulting in reduced repair of UV-induced DNA damage [18, 19]. We also identified that melanoma tumours with low *XPC* expression have significantly shorter survival [18]. The functional loss of NER in melanoma has also been reported by Belanger et al. [20] and could account for the high UV mutation signature of melanoma. This was further supported by analysis of melanoma genomes, that concluded somatic mutations active gene promoters is caused by a decrease in the levels of nucleotide excision repair (NER) activity [21, 22]. We have also shown that these same GGR transcripts do not respond to the platinum agent cisplatin in melanoma compared to normal melanocytes, which may be responsible for resistance to this treatment [23].

A role for GGR in melanoma development and chemotherapy resistance may come from the broad functions it has in controlling the DNA damage response. Damage recognition by XPC and DDB2 leads to activation of other pathways that control cell cycle and apoptosis, in addition to NER. XPC and DDB2 are involved the activation of the checkpoint signalling protein ATR in response to UV-induced DNA damage [24]. Both proteins also play a role in apoptosis in response to DNA damage [25, 26]. Additionally, XPC deficient cells have a significantly reduced cisplatin-mediated p53 and apoptotic response [27, 28], suggesting that DNA damage recognition is an important part of cisplatin induced apoptosis. Therefore, loss of GGR, in particular XPC, in melanoma could play a role in resistance to platinum chemotherapies.

The underlying mechanism that is responsible for the GGR deficiency seen in melanoma is yet to be identified. To date, somatic mutations in XPC, DDB1 and DDB2 have rarely been reported in melanoma tumours. We reported that upstream regulators of GGR including p53, BRCA1 and PCNA are not responsible [18, 23, 29]. One possible mechanism affecting the expression of these genes is dysregulation of epigenetics such as DNA methylation. Aberrant changes in DNA methylation patterns are a key feature of many cancers including melanoma, where global hypomethylation increases DNA instability and local hypermethylation of promoter CpG islands can silence the expression of many tumour suppressor genes [30]. DNA methylation is one of the best studied epigenetic modifications and has high potential in cancer research as a target due to DNA methyltransferase inhibitors such as decitabine (5-aza-2’deoxycytidine) that can demethylate and reverse silencing of genes [31].

To date there has only been only one study to investigate the methylation of XPC in melanoma. A mouse model found that melanocytes with BRAF^V600E^ and p14ARF^−/−^ background developed melanoma in response to UV radiation, with impaired DNA repair capacity due to reduced XPC expression from promoter hypermethylation [32]. However, as this study only examined the methylation of three CpG sites within the XPC promoter, further investigation is warranted.

More recently, the importance of CpG island shore methylation altering the expression of genes in cancer [33], has been reported. Methylation patterns within the CpG island shores of XPC have not been investigated. Methylation in these regions has a strong effect on the expression of genes and several studies have now identified changes altering expression of genes in various cancers [34–36].

As there is evidence of silencing of XPC by methylation in melanoma the aim of this study was to investigate the methylation pattern of the XPC promoter region, including the CpG island and flanking shores, and its effect on gene expression in our melanoma cell lines that display reduced GGR. We also examined if methylation patterns could be altered by demethylation and restore XPC function, therefore reinstating platinum chemotherapy sensitivity.

## Methods

### Cell culture and treatment

Four melanoma cell lines were used in this study: MM200, Sk-mel-28, Me4405 and Mel-RM. The source, tumour status and p53 status of each cell line has been previously described [37–39]. A human neonatal, medium pigment HEMn-MP melanocyte cell line was purchased (Cascade Biologics, USA and ThermoFisher, USA). Cell lines were authenticated as previously described [23] using GenePrint 10 (Promega). Mycoplasma was tested and not detected at 6 month intervals using MycoSEQ mycoplasma detection kit (Thermo Fisher Scientific). Melanoma cell lines were cultured in high glucose DMEM (5% FBS) (Gibco, Thermo Fisher Scientific) and HEMn-MP was cultured in Medium 254 (Gibco, USA). All cells were incubated at 37 °C 5% CO_2_ (Hera Cells 240, Thermo Scientific).

Carboplatin (Sigma-Aldrich) and decitabine (5-aza-2′-deoxycytidine) (Sigma-Aldrich) were resuspended in MilliQ H_2_O at 10 mg/mL and 1 mM respectively, with decitabine stored at − 80 °C. For treatment decitabine was diluted in cell culture medium to either 10 μM or 0.26 μM where indicated. Cell lines were treated with decitabine for 72 h with cell culture media replaced every 24 h with fresh media and decitabine. Carboplatin was diluted in cell culture medium to 8 μg/mL and cells treated for 48 h. For combination treatment cell lines were treated first with 0.26 μM decitabine for 72 h followed by 48 h of 8 μg/mL carboplatin. These doses were chosen as they were based on plasma concentrations of each drug when used as chemotherapy agents [40, 41].

### Global DNA methylation quantification

Global DNA methylation levels were quantified using a 5-mC DNA ELISA Kit (Zymo Research) as per manufacturer’s instructions. DNA from melanoma cell lines before and after decitabine treatment was extracted by Quick-DNA Universal Kit (Zymo Research). 100 ng of genomic DNA and methylated standards were bound to an ELISA plate and methylated DNA was detected with antibodies to 5-methylcytosine, quantified by colorimetric analysis.

### Gene expression analysis

Before and following treatment at specified time points RNA was collected by phenol chloroform extraction. RNA (1 μg) was reverse transcribed in triplicate using the High Capacity Reverse Transcription Kit (Thermo Fisher Scientific) and the resultant cDNA was diluted 1:20 as previously described [23]. Relative expression of *XPC* was measured in triplicate and normalised to the geometric mean of three housekeeping genes, *GAPDH*, *ACTB* and *18S rRNA* using TaqMan gene expression assays and a Viia7 system (Applied Biosystems). Relative expression was calculated using 2^-ΔCt^.

### Western blotting

Nuclear protein fractions were obtained using the NucBuster protein extraction kit (Merck Millipore). Protein lysate (40 μg) was added to 4X SDS-sample loading buffer (250 mM Tris-HCl, pH 6.8, 4% LDS, 40% (*w*/*v*) glycerol, 0.02% bromophenol blue, 15% beta-mercaptoethanol) and denatured by boiling for 5 min. Samples were loaded onto 4-20% TGX precast polyacrylamide gels (Bio-Rad Laboratories) and run at 150 V (constant voltage) in Tris-Glycine buffer (25 mM Tris, 192 mM glycine, 0.1% SDS). Proteins were transferred onto nitrocellulose using the TransBlot Turbo system (high-MW 10 min; Bio-Rad Laboratories) and visualised using Ponceau S (0.1% (*w*/*v*) Ponceau S in 5% acetic acid; Sigma-Aldrich). Following transfer, blots were blocked in 5% skim milk for 1 h at room temp. XPC was detected using anti-XPC rabbit polyclonal antibody (H-300) (1:200; sc-30,156 Santa Cruz Biotechnology, Inc.) and anti-TATA binding protein (TATA-BP) (1:1000 ab51841, Abcam) was used as a nuclear loading control. Primary antibodies were incubated at 4 degrees overnight. Blots were washed three times for 5 min in PBS-T then incubated for 1 h at room temperature with HRP-conjugated secondary antibodies (goat anti-rabbit 170-6515, Bio-Rad Laboratories). Blots were washed as done previously then proteins detected by chemiluminescence using Clarity Western ECL reagent (Bio-Rad) and imaged using the ChemiDoc MP system (Bio-Rad Laboratories). Image processing and densitometry analysis was performed on all blots using ImageJ. Data was normalised to TATA-BP and expressed as fold induction from baseline.

### Bisulfite sequencing of XPC

DNA was bisulfite converted using an EZ DNA Methylation Kit (Zymo Research) according to manufacturer’s instructions. The CpG island and surrounding shores of *XPC* promoter was amplified by PCR using Taq Polymerase (Invitrogen) and the primers (Additional file 1: Table S1). All PCRs were performed in triplicate for all cell lines before and after treatment with 0.26 μM decitabine. PCR products were cleaned with Exonuclease I and Alkaline Phosphatase (Thermo Fisher Scientific). For sequencing, fragments for each sample were pooled and libraries prepared using the TruSeq Nano DNA Library Prep kit (Illumina). Sequencing was performed on an Illumina MiSeq and analysed using Bismark [42].

### Flow cytometry

After treatment with 0.26 μM decitabine, 8 μg/ml carboplatin and in combination, both attached and detached cells were collected. Apoptotic cells were quantified after drug treatment using an Annexin V Apoptosis Detection Kit (BD Biosciences) following manufacturers instruction performed on a BD FACSCanto II flow cytometer (BD Biosciences). 1 × 10^6^ cells before and after treatment were washed and stained with 7-AAD and PE conjugated Annexin-V for 15 min in the dark. Data was analysed on FlowJo v10 (FlowJo, LLC). Apoptotic cells were quantified as the percentage of cells that stained positive for Annexin-V and double Annexin/7-AAD positive cells.

### Cell proliferation and senescence detection

Cellular proliferation after treatment was measured using a CellTitre-Glo Luminescent Cell Viability Assay kit (Promega) according to manufacturer’s instructions. Cells were seeded in 96-well plates at 5 × 10^3^ cells per well overnight before drug treatment and luminescence measured on a Cytation 3 plate reader (BioTek). After combination treatment senescence was measured by β-galactosidase staining using an Abcam Senescence Detection Kit (Abcam). Cells were plated and fixed in 6-well plates after combination treatment and stained with X-gal. Positively stained cells were identified under a light microscope.

### siRNA knockdown

The expression of *XPC* was knocked down after decitabine treatment using siRNA purchased from Dharmacon (siGENOME Human XPC, D-016040-04-0010). Transfections were carried out in the last 24 h of decitabine treatment with 25 nM of *XPC* siRNA in OptiMEM medium (Gibco) using Lipofectamine RNAiMAX (Invitrogen) according to manufacturer’s instruction. An NTC siRNA (siCONTROL Non-targeting siRNA #4, Dharmacon) was used as a control for transfection in identical conditions to *XPC* siRNA.

### Statistical analysis

Statistical analysis was performed using GraphPad Prism 6 (GraphPad Software). Non-parametric Mann-Whitney tests were used to assess differences between groups. A *p*-value of < 0.05 was considered statistically significant.

## Results

### Decitabine can demethylate melanoma and increase *XPC* expression

**Fig. 1:**
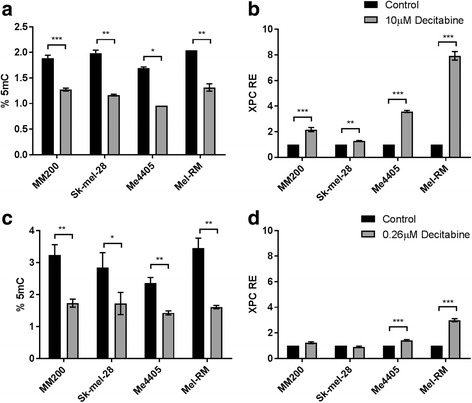
Global methylation levels and *XPC* expression in melanoma after decitabine treatment. Melanoma cell lines were treated with 10 μM decitabine (**a**) or 0.26 μM decitabine (**c**) (grey) for 72 h and global methylation levels (%5mC) were quantified and compared to untreated cells (control, black). *XPC* transcript expression (RE) after 10 μM decitabine (**b**) and 0.26 μM decitabine (**d**) was quantified by qPCR and normalised to control. Data represent mean of triplicate experiment, bars = SEM. *p < 0.05, **p < 0.01, ***p < 0.001

As an initial test to determine global methylation levels in melanoma, cell lines MM200, Sk-mel-28, Me4405 and Mel-RM were treated with the demethylating agent decitabine. Cells were treated with either 10 μM or 0.26 μM decitabine and global DNA methylation levels (%5mC) and *XPC* relative expression (RE) were quantified in response (Fig. 1). Treatment with 10 μM decitabine significantly (MM200 *p* = 0.0004, Sk-mel-28 *p* = 0.003, Me4405 *p* = 0.02, Mel-RM *p* = 0.002) reduced methylation levels in all melanoma cell lines with an average reduction of 38.22% ±4.98 (Fig. 1a). This corresponded with highly significant (MM200, Me4405, Mel-RM *p* < 0.0001, Sk-mel-28 *p* = 0.0008) increases in *XPC* mRNA expression in all cell lines (1.27-7.93 fold increase) (Fig. 1b).

A lower, pharmacologically relevant dose of decitabine (0.26 μM) was also tested. Lower doses of decitabine limits the formation of DNA damage and cytotoxicity but can still demethylate [43]. 0.26 μM decitabine also significantly demethylated all melanoma cell lines (MM200 *p* = 0.005, Sk-mel-28 *p* = 0.03, Me4405 *p* = 0.002, Mel-RM *p* = 0.001) (Fig. 1c) with an average of 44.67% ±6.69. However, at this dose only two of the four melanoma cell lines had a significant increase in *XPC* mRNA expression (1.23-2.99 fold) (Me4405 *p* = 0.0003, Mel-RM *p* < 0.0001) (Fig. 1d), which was lower than the increase seen with 10 μM. Taken together, this data shows that global demethylation with decitabine does occur and can increase *XPC* mRNA expression in melanoma. Due its clinical relevance, all further experiments were performed with 0.26 μM decitabine.

### XPC promoter methylation patterns in melanoma

**Fig. 2:**
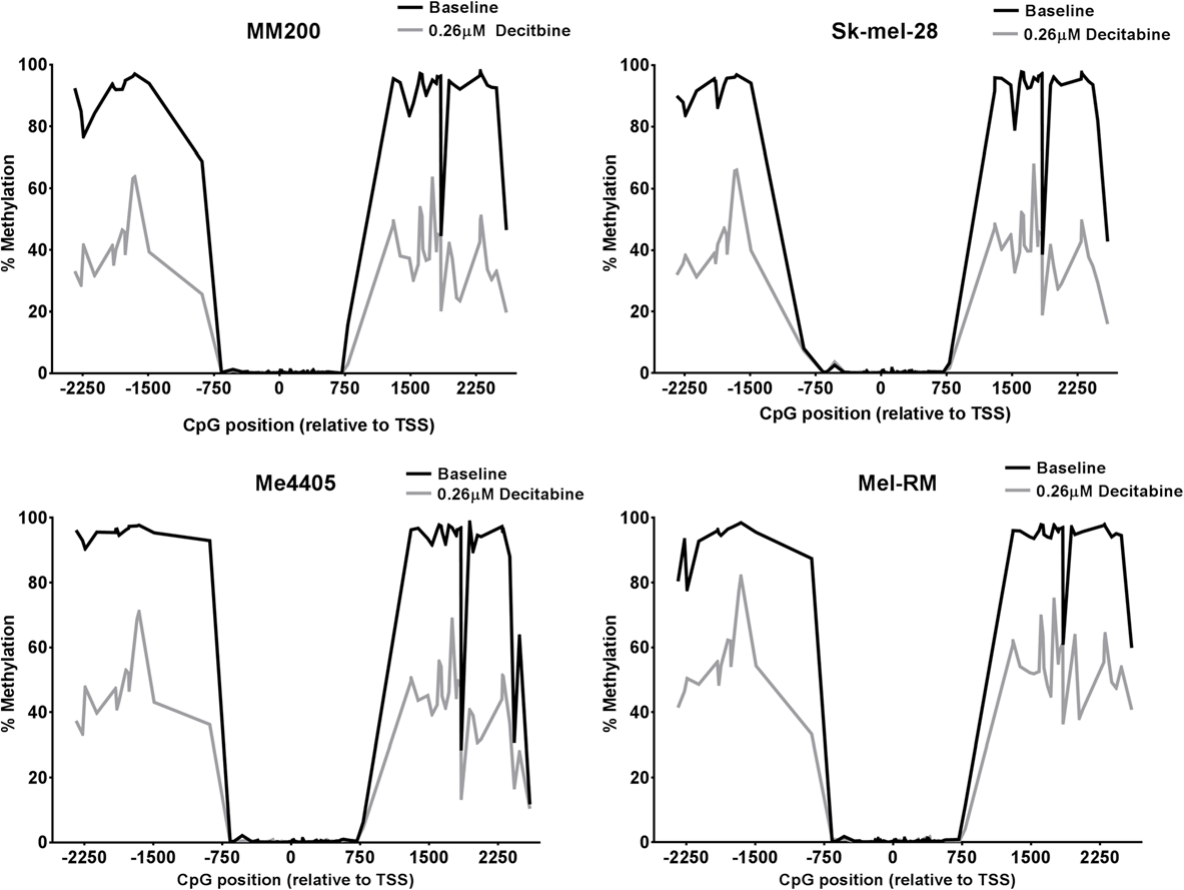
DNA methylation pattern of the XPC CpG island before and after decitabine. Methylation levels in each melanoma cell line at baseline (black) and after treatment with 0.26 μM decitabine (grey) was quantified by bisulfite sequencing. CpG position is shown relative to XPC transcription start site (TSS). Upstream (5′) shore = position − 2341 to − 423, CpG island = position − 364 to 568, Downstream (3′) shore = position 714 to 2596

As demethylation increased *XPC* mRNA expression in melanoma, the promoter region of *XPC*, containing the CpG island and adjacent shores, was bisulfite sequenced in all melanoma cell lines before and after decitabine treatment to identify if promoter methylation is responsible for reduced expression. The XPC promoter region was sequenced by next generation bisulfite sequencing allowing for quantification of methylation at base resolution (Fig. 2). Percent methylation at each CpG site was quantified by aligning the bisulfite converted sequence and calculating percent methylation based on C or T using the Bismark software package [42].

The CpG island of *XPC* had very low methylation in all cell lines, less than 1.5%. As high methylation of the CpG island is associated with gene silencing, these very low levels of methylation in melanoma here implies that methylation of the CpG island in the *XPC* promoter is not responsible for reduced *XPC* expression. The upstream (5′) shore showed high levels of methylation (average 91.8%) with the exception of the four CpG sites closest to the island which were methylated approximately 0-5%. The downstream (3′) shore showed a similar pattern, of high methylation, to the upstream shore. While most sites in the shores were consistently highly methylated, several sites varied in methylation levels across the melanoma cell lines. For example, the CpG site 1849 bp from the TSS displayed methylation between 28 and 61% in melanoma cell lines. Similarly, the last four CpG sites in the shore had reduced methylation in Me4405 where methylation was as low as 11% (Fig. 2).

These methylation patterns are consistent with data from the Cancer Genome Atlas (TCGA) (http://cancergenome.nih.gov/) which contains methylation data for 470 melanoma tumours. Although the TCGA dataset data set was collected using the Infinium HumanMethylation450 array it only covers 21 CpG sites across the *XPC* CpG island and 2 within one shore. Each of these sites showed similar methylation pattern as our sequencing data.

Sequencing showed that the entire length of both shores were demethylated by 0.26 μM decitabine (Fig. 2). The downstream shore demethylated more than the upstream shore with an average loss of 43.2% methylation (MM200 = 48.06%, Sk-mel-28 = 46.49%, Me4405 = 41.88%, Mel-RM = 36.38%). The upstream shore had an average loss of 35.92% methylation (MM200 = 38.52%, Sk-mel-28 = 36.93%, Me4405 = 38.22%, Mel-RM = 30.02%). The pattern of methylation after decitabine treatment appears almost identical in all melanoma cell lines, indicating that some CpG sites are more susceptible to demethylation than others. For example two CpG sites at − 1656 and − 1678 ranged from 63.18-81.97% methylated after demethylation while the surrounding sites were demethylated to as little as 35% methylation, forming a peak in the upstream shore. Similar peaks are evident in the downstream shore in all cell lines suggesting that demethylation in the shores is not random. As such, no remarkable pattern of methylation in *XPC* is able to explain why 0.26 μM decitabine increases expression of *XPC* in Me4405 and Mel-RM but not MM200 or Sk-mel-28. Further stimuli may be needed to induce expression in these non responsive cell lines.

### XPC is induced in melanoma by carboplatin after decitabine treatment

**Fig. 3:**
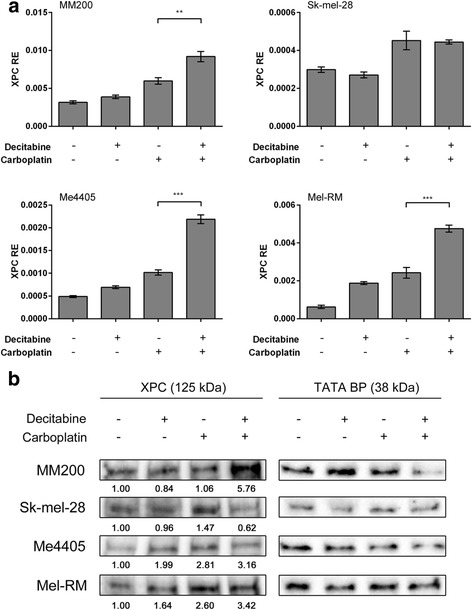
Combined decitabine and carboplatin treatment induces XPC expression in melanoma. Melanoma cell lines were treated with 0.26 μM decitabine for 72 h, 8 μg/mL carboplatin for 48 h, or both in sequential combination. *XPC* expression was quantified by qPCR in response to single and combined treatments (**a**). Baseline expression with no treatment was used as a control. Significance displayed between carboplatin alone and combination treatment. Data represent mean of triplicate experiment, bars = SEM. **p < 0.01, ***p < 0.001. Western blot (**b**) of XPC after single and combined decitabine (0.26 μM) and carboplatin (8 μg/mL) treatment. Numbers represent fold change from baseline. Data was normalised to loading control (TATA BP)

As the lower dose (0.26 μM) still demethylated but did not increase *XPC* expression as significantly as 10 μM, we investigated if *XPC* expression is induced in response to DNA damage caused by the platinum chemotherapy agent carboplatin, after demethylation. Melanoma cell lines were treated with decitabine (0.26 μM) and carboplatin (8 μg/mL), both individually and in sequential combination and the expression of *XPC* was measured (Fig. 3a). Carboplatin alone resulted in small increases in *XPC* expression in three melanoma cell lines (MM200, Me4405 and Mel-RM). When decitabine is used to demethylate before carboplatin treatment, the increase in *XPC* expression is significantly greater, increasing the fold change from 1.52-3.86 (carboplatin alone) to 1.49-7.55 fold increase (decitabine and carboplatin). With the exception of Sk-mel-28, the level of *XPC* expression after combination treatment was significantly higher than carboplatin alone (MM200 *p* = 0.001, Sk-mel-28 *p* = 0.25, Me4405 *p* < 0.0001, Mel-RM p < 0.0001). This suggests that demethylation, while not consistently affecting baseline expression of XPC, can lead to a greater induction of *XPC* in response to DNA damaging agents such as carboplatin.

The increased expression of XPC in response to combination treatment was confirmed at the protein level (Fig. 3b). Three of the four cell lines had greater expression of XPC protein after sequential decitabine and carboplatin (3.16-5.76 fold increase from baseline). As with mRNA, Sk-mel-28 did not have a great induction of XPC from combined treatment compared to carboplatin alone.

### Decitabine increases sensitivity to carboplatin induced cell death

**Fig. 4:**
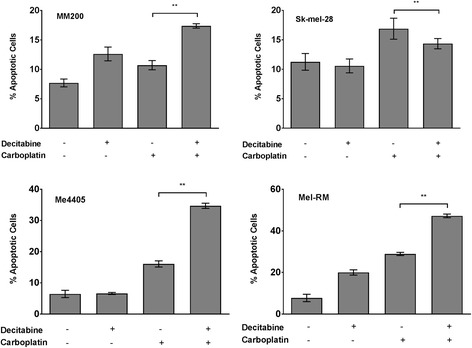
Pre-treatment with decitabine enhances susceptibility to carboplatin cytotoxicity. Apoptotic melanoma cells after treatment with 0.26 μM decitabine, 8 μg/mL carboplatin or both in sequential combination was quantified in melanoma cell lines by flow cytometry. Baseline with no treatment was used as a control. Data represents mean of triplicates of three individual experiments, bars = SEM. **p < 0.01

To investigate if the increase in XPC expression following demethylation has a functional consequence on the cytotoxic response to carboplatin, apoptosis was quantified following single and combination treatment (Fig. 4). Figure 4 shows cells undergoing apoptosis, as marked by Annexin V staining, as result of drug treatment quantified by flow cytometry. Baseline levels of apoptosis ranged from 6.5% to 11.3% which is consistent with previous reports for Sk-Mel-28 [44, 45] MM200 [44, 45] Mel-RM [45, 46] and me4405 [45] cell lines. Decitabine alone triggered an apoptotic response in both MM200 and Mel-RM, shown by the increase in apoptotic cells and this response was amplified greatly by following decitabine with carboplatin (1.6 fold). While Me4405 did not show an increase in apoptosis to decitabine alone, a strong induction occurred in response to combination treatment (2.2 fold).

The cytotoxic potential of combined decitabine and carboplatin treatment is seen where combined treatment resulted in significantly higher levels of cell death in three out of the four cell lines (MM200, Me4405, and Mel-RM) when compared to carboplatin alone (Fig. 4). Interestingly, Sk-mel-28 did not show greater levels of apoptosis for combination treatment and this was the only cell line where treatment did not induce expression of XPC. Altogether, this data shows that combining decitabine and carboplatin induces a greater apoptotic response in the majority of these melanoma cell lines.

### Combination of decitabine and carboplatin decreases melanoma cellular proliferation

**Fig. 5:**
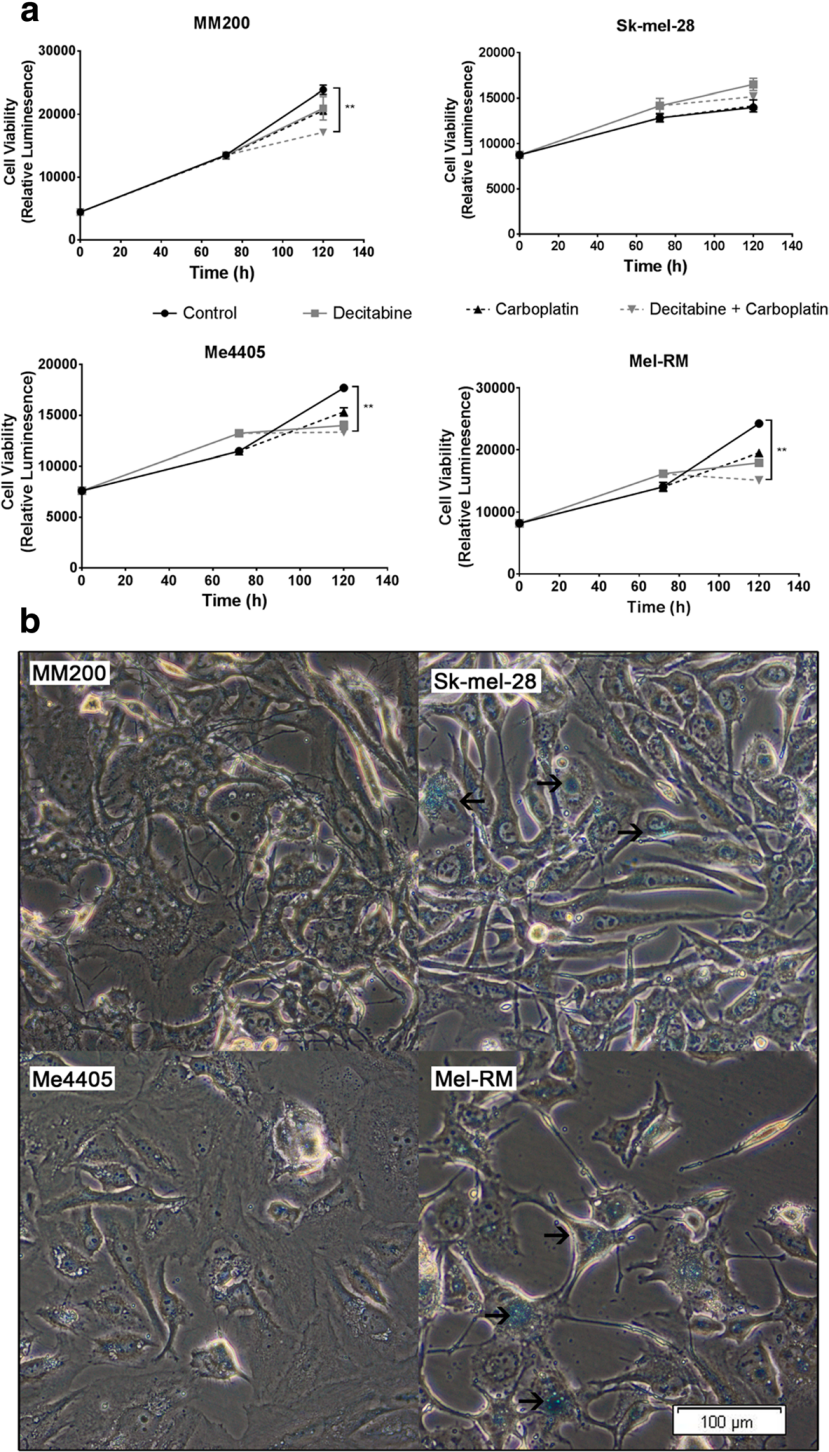
Combined decitabine and carboplatin decreases melanoma proliferation and can induce senescence. **a** Growth rate of melanoma cell lines treated with a control (DMEM), 0.26 μM decitabine, 8 μg/mL carboplatin or combined treatment. Data displayed is mean of triplicate experiments, error = SEM, significance compared between combined decitabine and carboplatin, and control, **p < 0.01. **b** Representative bright-field microscopy image of senescence associated β-galactosidase staining in all four melanoma cell lines after combined decitabine and carboplatin treatment. Arrows indicate regions of positive staining, bar = 100 μm

As not all melanoma cell lines had an increased apoptotic response to combined decitabine and carboplatin, cellular proliferation was measured to see if cell growth was affected by combination treatment (Fig. 5a). In the first 72 h of treatment cells treated with decitabine (grey) and DMEM control (black) grew at a similar rate in all cell lines. At 72 h, DMEM control (solid lines) or carboplatin (broken lines) was added to both groups. As expected, all cell lines, with the exception of Sk-mel-28, treated only with DMEM continued to proliferate at a steady rate over the next 48 h (solid black). When treated with decitabine only (solid grey) or carboplatin only (broken black) all cell lines, again with the exception of Sk-mel-28, continued to proliferate although at a slower but non-significant rate compared to DMEM control. Only the combination of decitabine and carboplatin (broken grey) significantly slowed the growth of the melanoma cell lines (Fig. 5a). This suggests that the cells which are undergoing apoptosis in response to combination treatment, also have significantly slowed proliferation.

To identify if this response is just a decrease in the rate of proliferation or if the melanoma cells are being driven into senescence, cell lines were stained with a senescence detection kit after combination decitabine and carboplatin treatment (Fig. 5b). Two of the melanoma cell lines (Sk-mel-28 and Mel-RM) showed positive β-galactosidase staining, a marker of senescence in response to combination treatment (highlighted by arrows), while cell lines MM200 and Me4405 did not stain positive (Additional file 2: Fig. S1). Altogether, these results show that combination decitabine and carboplatin significantly reduces the rate of growth of melanoma cells, with some cell lines being driven into senescence in response.

### Effects of combination decitabine and carboplatin are partially dependent on *XPC* expression

**Fig. 6:**
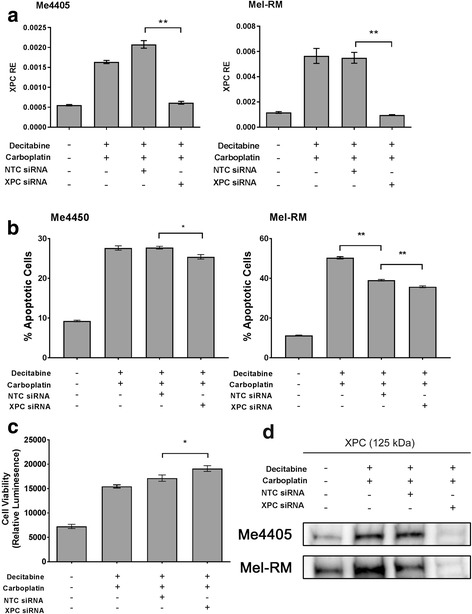
XPC knock down has small but significant impact on combined treatment. Melanoma cell lines Me4405 and Mel-RM were treated with combined decitabine (0.26 μM) and carboplatin (8 μg/mL) in the presence of XPC siRNA or non-targeting control (NTC). Knockdown of XPC was confirmed at the transcript by qPCR (**a**) and protein level by western blot (**d**). Apoptosis (**b**) was quantified to identify the effect of XPC knockdown on the response to combined treatment. Mean cell proliferation (**c**) for Me4405 and Mel-RM was quantified to further examine the effect of XPC knockdown on the response to combined treatment. Data represents mean of triplicate experiments, bars = SEM, *p < 0.05, **p < 0.01

To identify whether the response to the combination treatment is dependent on the increased XPC expression after demethylation, cell death and proliferation experiments were repeated while XPC expression was knocked down using siRNA (Fig. 6). XPC siRNA was added to cell culture in two cell lines (Me4405 and Mel-RM) for the last 24 h of the 72 h decitabine treatment to counter the increase in XPC expression while not effecting the expression of any other gene upregulated by global demethylation. Figure 6a shows that the XPC siRNA significantly reduces the expression of *XPC* after combination treatment compared to non-targeting control (NTC), to a level similar to baseline. This is also reflected in the expression of XPC protein (Fig. 6d). Reduction of XPC resulted in a small but significant (Me4405 *p* = 0.01, Mel-RM *p* = 0.002) decrease in the number of apoptotic cells in both Me4405 and Mel-RM (1.09 fold) after combination treatment (Fig. 6b). Knock down of XPC also affected the proliferation of cells after treatment. Cells treated with combination treatment without the increased expression of XPC (XPC siRNA) had a significantly higher level of viable cells (1.08 fold) (*p* = 0.027) after treatment compared to NTC control (Fig. 6c). XPC siRNA had no effect on the presence of β-galactosidase staining. Overall these results suggest that the effects of combination treatment are at least partially dependent on the increased *XPC* expression in melanoma cells.

## Discussion

We have previously reported that XPC does not respond to DNA damage in melanoma [18, 19, 23], which may be a key component of melanoma development from UV exposure and resistance to platinum chemotherapies. The cause of this loss has not been discovered, but some evidence exists of DNA methylation altering XPC expression [32]. In this study, we investigated the effect of DNA methylation on the expression of *XPC* in melanoma. Here we have shown that, while methylation may not be the cause of reduced XPC expression, treatment of melanoma cell lines with decitabine can restore expression, and allow for strong induction in response to carboplatin. The sequential treatment of melanoma cell lines with decitabine and carboplatin also increased apoptosis and decreased cell proliferation, suggesting that this combination can overcome platinum resistance in vitro.

Bisulfite sequencing revealed that the CpG island of the XPC promoter was not methylated in the cell lines. This methylation patterns in our study are consistent with data available from the Cancer Genome Atlas (TCGA) showing that the *XPC* CpG island is not methylated in melanoma tumours. The same pattern of methylation was also seen in a melanocyte cell line (Additional file 3: Fig. S2) suggesting it is a lineage-specific epigenetic pattern.

Methylation within the CpG island shores was present in melanocytes and the 4 melanoma cell lines in this study. The methylated regions were partially demethylated by decitabine in the melanoma cell lines. As melanocytes have low replication rates in vitro and do not replicate in vivo the blocking of methylation does not occur to the same extent as in melanoma cells, therefore the effect of decitabine is not seen in melanocytes. More research is needed to confirm that demthylation of the *XPC* CpG island shores is responsible for increased XPC expression in response to decitabine. It is possible that demethylation of the shores allows for some other elements such transcription factors access to *XPC* which may result in the increased expression in response to decitabine. One possible explanation is that an upstream regulator of *XPC* expression is also being demethylated by decitabine and can induce *XPC* expression.

Two doses of decitabine were used in this study. The first (10 μM) was chosen as it is a high dose that would ensure demethylation across the genome, while the second (0.26 μM) represented a pharmacological dose [40]. 10 μM decitabine induced a greater increase in *XPC* when compared with 0.26 μM. This may have been due to the fact that high doses of decitabine can induce DNA damage by prolonged binding of DNMT1 leading to double stranded breaks [47]. It could be that in combination with demethylation, this type of damage signalled for *XPC* upregulation. The lower dose of 0.26 μM should not have induced as great of levels of damage and as such could explain why only two cell lines Mel-RM and Me4405 increased *XPC* expression after 0.26 μM decitabine.

To identify if platinum-induced DNA damage would induce *XPC* following demethylation, we treated the cells with carboplatin following demethylation with 0.26 μM decitabine. Combined treatment resulted in a significantly greater *XPC* response in 3 of 4 cell lines. While low dose decitabine was not enough to increase *XPC* expression alone it may demethylate a particular region of *XPC* or an upstream target that then allows *XPC* to respond to the DNA damage signal caused by carboplatin.

CpG island shores have been gaining more consideration in the past few years after being confirmed as one of the major regions for differential methylation in cancer [33, 48]. Although the specific function of shore methylation has not yet been identified, changes in methylation are reported to affect expression of genes. The *HOX10* gene has CpG island shore methylation that is associated with transcriptional repression in breast cancer [36]. Methylation in the shores varied from 5 to 95% and was inversely correlated with expression; those with higher shore methylation had lower expression. A similar pattern was found in the caveolin-1 (*CAV1*) gene [34]. This study found a negative relationship between *CAV1* shore methylation and expression in breast cancer. This relationship has been found further in other genes in other cancers [49, 50]. All this data surrounding shore methylation indicates that shore methylation is associated with transcriptional repression but the exact molecular mechanisms of this relationship are not understood.

Regardless of the dynamics of *XPC* demethylation and expression, this study revealed some exciting results with translational potential. Demethylation with decitabine increased the sensitivity of melanoma cells to the growth inhibitory and apoptotic effects of carboplatin, which is typically ineffective in melanoma [51]. The cell lines that showed an increased XPC expression also had significantly higher levels of apoptosis and cell death. This was combined with a decreased rate of cell proliferation, and senescence in some cell lines. These results were much greater than those compared to carboplatin alone. Sk-mel-28 did not induce *XPC* or apoptosis in response to decitabine and carboplatin. Sk-mel-28 carries a mutation that results in constitutively activated p53 [52] which may account for the lack of response, but requires further investigation. Carboplatin is a slower acting drug compared to other platinum chemotherapies such as cisplatin and does not induce as much DNA damage [53]. This means an even greater response could be seen if a second carboplatin dose was added after demethylation.

The importance of *XPC* in the response to combined treatment was investigated. When *XPC* was knocked down with siRNA during decitabine treatment minor changes were seen in apoptosis and proliferation. Given the effect of XPC knockdown on apoptosis was minor, despite robust attenuation of XPC, the effect of exogenous overexpression of XPC expression is required to further clarify if the effects that are being observed are indeed related to XPC expression.

The data reported herein suggests *XPC* is not the only driver of the responses seen. This could be expected as there are numerous and complex pathways that are involved in cell death and proliferation, which combined with the fact decitabine demethylates globally, may lead to many changes in various pathways. It is unlikely that one single demethylated target will drive the response alone. The melanocyte-lineage specific master regulator, MITF may additionally contribute to the apoptotic response to decitabine and carboplatin. Although this requires further investigation, MITF is involved in melanoma proliferation and survival (reviewed in [54]) and has been associated with DNA repair in melanoma [55]. Regardless of the role of the *XPC* response and its importance, here we have identified a potential combination treatment in melanoma.

While some studies have examined the effect of decitabine on melanoma growth and apoptosis, ours is the first study to examine the combination of demethylation with platinum therapy within melanoma. A study in 2011 showed that decitabine has a cytotoxic effect on melanoma that is independent of apoptosis [56]. While apoptosis remained low from treatment, decitabine induced G2/M cell cycle arrest, inhibiting the growth of a melanoma cell line. Decitabine was also able to induce differentiation, marked by the formation of melanocyte-like dendritic structures. Similar results are seen in another study that expands treatment to a panel of cell lines and a mouse xenograft model [57]. In response to decitabine the majority of cell lines showed reduced proliferation and markers of melanocytic differentiation, including dendritic formation, decreased nuclear-cytoplasmic ratio, and increased melanin production. Again, apoptosis was not a significant response to decitabine treatment. The response was discovered to be independent of p53 and *CDKN2A*. Differentiation was driven by demethylation of the melanocyte differentiation driver *SOX9* promoter and subsequent increased expression. Exit from the cell cycle and reduced proliferation was a result of upregulated p21 (*CDKN1A*), and p27 (*CDKN1B*). Whole transcriptome analysis in response to decitabine [58] confirms a role for p21 and proposes a role for the WNT signalling and β-catenin pathway in both resistance to decitabine and supressing apoptosis from decitabine treatment alone.

While a new idea in melanoma, combining demethylation treatment with platinum chemotherapies has been studied in other cancer types with positive results. One particular study examining multiple combinations found that decitabine had the greatest combination potential with platinum compounds, out of a screen of 16 drugs [59]. Decitabine can also rescue cisplatin resistance in head and neck squamous cell carcinoma (HNSCC) by demethylating of panel of genes associated with HNSCC resistance, and enhancing the cytotoxic and apoptotic effects of cisplatin [60]. This combination is also showing promise in ovarian cancer tumours. One study showed combined treatment resulted in a high response rate and progression free survival in platinum resistant patients [61]. In this study pathways enriched for demethylation include pathways in cancer, WNT signalling, and apoptosis, and some demethylated genes such as *HOXA10* and *RASSF1A* correlated with progression free survival. Similar results have been found in neuroblastoma [62], renal carcinoma [63] and non-small cell lung cancer [64]. This makes combined decitabine and carboplatin treatment worthy of investigation in melanoma as it shows potential in other platinum resistant cancers.

The benefits of a combination decitabine and carboplatin treatment are not limited to DNA damaging and apoptotic mechanisms. One major challenge in cancer treatment is the ability of tumours to escape from immune detection and prevent efficient T-cell response to cancer cells [65]. Emerging research shows both carboplatin and decitabine may also have therapeutic benefits by enhancing the immunogenicity of tumour cells, allowing the immune system to target the cancer. This offers an interesting potential to be combined as a priming regime for immunotherapy.

Platinum chemotherapy compounds including carboplatin can induce a combination of stress and cell death that can initiate a tumour-specific immune response. Firstly, platinum therapy induces endoplasmic reticulum (ER) stress that results in calreticulin exposure on the cell surface, acting as a signal for dendritic cells [66]. Secondly, cells undergoing apoptosis from platinum compounds release ATP which acts as a chemoattractant for dendritic cells and macrophages to the tumour site [67]. Thirdly, during cell death, high mobility group protein 1 (HMGB-1) is released from the nucleus. This binds to toll like receptor 4 (TLR4) on dendritic cells leading to cytokine secretion and cross presentation [68]. Altogether these factors result in the attraction and maturation of dendritic cells. Additionally drugs such as carboplatin, but not other chemotherapies, have been shown to downregulate the programmed death ligand 2 (PDL-2) on both dendritic cells and tumour cells, resulting in enhanced antigen specific T-cell activation, through the IL-4/STAT6 pathway [69]. This can result in tumour antigen presentation in the lymph nodes leading to increased activated tumour-specific T-cells which will mount an immune response against the tumour [70].

## Conclusion

Taken together the results of this study indicate treatment of melanoma with decitabine can sensitise cells to sequential carboplatin treatment. Demethylation can restore *XPC* induction in response to DNA damage. Demethylation of CpG island shores is likely to be responsible for this, but further research is needed to confirm this finding. Regardless, we have discovered the potential of a combination treatment for melanoma using decitabine to re-instate carboplatin sensitivity resulting in greatly increased apoptosis and decreased cellular proliferation. Further research into how this combination affects methylation genome wide in melanoma is needed to completely elucidate the mechanisms leading to this outcome.

## Additional files

Additional file 1: Table S1. XPC bisulfite promoter primers for PCR. (DOCX 14 kb)

Additional file 2: Figure S1. Representative bright-field microscopy images of senescence associated β-galactosidase staining in all four melanoma cell lines after combined decitabine and carboplatin treatment. Arrows indicate regions of positive staining, bar = 100 μm. (TIFF 42085 kb)

Additional file 3: Figure S2. DNA methylation pattern of the XPC CpG island in melanocytes and melanoma. Methylation levels in melanocytes (black) and each melanoma cell line at baseline (grey) was quantified by bisulfite sequencing. CpG position is shown relative to XPC transcription start site (TSS). Upstream (5′) shore = position − 2341 to − 423, CpG island = position − 364 to 568, Downstream (3′) shore = position 714 to 2596. (TIFF 603 kb)

## Abbreviations

5-mC: 5-methylcytosine
ACTB: β-actin
CTA: Cancer testis antigen
DDB1: DNA damage binding protein 1
DDB2: DNA damage binding protein 2
GAPDH: Glyceraldehyde 3-phosphate dehydrogenase
GGR: Global genome repair
HLA: Human leukocyte antigen
HNSCC: Head and neck squamous cell carcinoma
NER: Nucleotide excision repair
NTC: Non-targeting control
siRNA: small interfering RNA
TATA-BP: TATA-binding protein
TCGA: The Cancer Genome Atlas
TCR: Transcription coupled repair
UV: Ultraviolet Radiation
XPC: Xeroderma Pigmentosum complementation group C
