# Supplementary material for: Sequential decitabine and carboplatin treatment increases the DNA repair protein XPC, increases apoptosis and decreases proliferation in melanoma
Source: BMC Cancer. 2018 Jan 26;18:100. doi: 10.1186/s12885-018-4010-9 (PMC5787239; doi:10.1186/s12885-018-4010-9)
Supplement: Supplementary file 1 — XPC bisulfite promoter primers for PCR. (DOCX 14 kb) [file 12885_2018_4010_MOESM1_ESM.docx]

Additional file 1: Table S1. XPC bisulfite promoter primers for PCR

| Region | Amplicon | Forward Primer | Reverse Primer |
| --- | --- | --- | --- |
| 5’ CpG Island Shore | 1 | ATTAGTTAGAATGTTAAATAGAGGTGTAGG | ACATAATTTTTAATCTCAATTCATAAATAT |
|  | 2 | AAGGTAAGTAAGTATTGGTTTTGGAGTTA | CCCAAAATACTAAAATTCAAAC |
|  | 3 | AAAAATAGTGTTAGGAAAATAGAAAATATT | CCTATCTCAACCTCCCAAATAACTA |
|  | 4 | TGAGATTAGTTTGGTTAATATGGTGAA | TCAAAACCTATTACCAATTCAATTACC |
|  | 5 | TTTTTTATGAAGAAAAATGAGGTAGG | CCTCATAAAACTATCAAAATTCCAATAA |
| CpG Island | 6 | TTTTGGTTTTTAGAATAGTGTTTAATTATA | TTTTTCCAAACTCAAAAAAAATAAC |
|  | 7 | TAATAGGAAAAGGTTTTAGGGTTTA | TACTTATCTAAACAAATTCCACTTC |
|  | 8 | GAGAAATTTGTTGTGTTTTTTTT | CCTACCTCTAAACCTCCTCC |
|  | 9 | GAAATTTAAGGTTAAGAGTAAGGTT | AAATCAACACACTACATCACTCTAC |
| 3’ CpG Island Shore | 10 | TTTAGAGGATATAGATGTTTAGGGTTGA | AAAAAAACTACAATCACTCAACTAAAAAAT |
|  | 11 | TTTTTTTATTTTTTTAAAATTTAAGTTAGA | AACCCTACATCCACAAATTCAAC |
|  | 12 | TGAATTTGTGGATGTAGGGTT | AAAAAACCCAAAAATTCCTTCTAC |
|  | 13 | TTGGTTAGGTTGGTTTTAAATTTTT | AATCCTCCAAAATATATTTATCTCTCATAT |
|  | 14 | TGTTGATATGATATTTATATGTAAAATATT | TAAACTTAAATACATCTTTACCTCC |
