# Supplementary figures and images for: Sequential decitabine and carboplatin treatment increases the DNA repair protein XPC, increases apoptosis and decreases proliferation in melanoma
Source: BMC Cancer. 2018 Jan 26;18:100. doi: 10.1186/s12885-018-4010-9 (PMC5787239; doi:10.1186/s12885-018-4010-9)

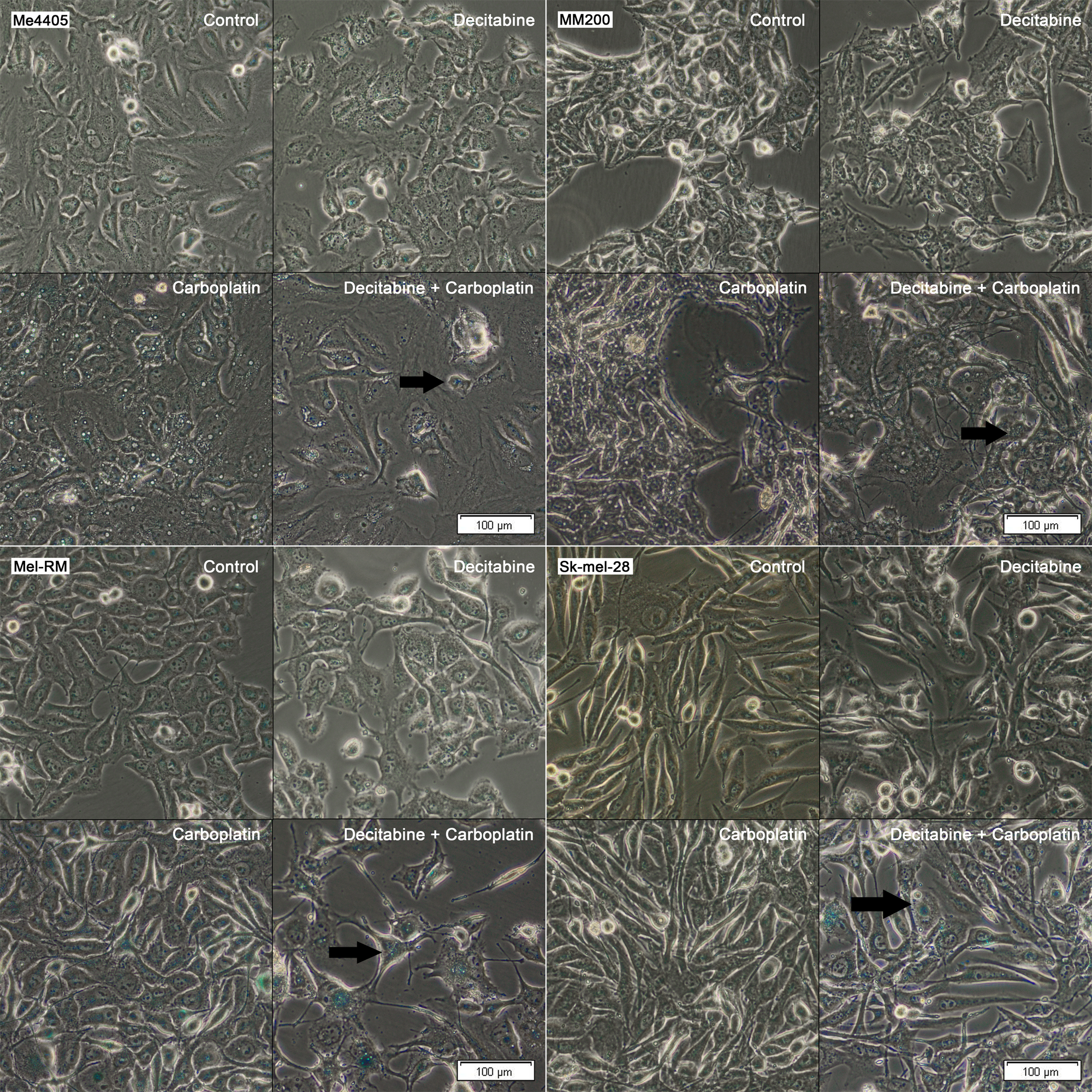

Supplement: Supplementary file 2 — Representative bright-field microscopy images of senescence associated β-galactosidase staining in all four melanoma cell lines after combined decitabine and carboplatin treatment. Arrows indicate regions of positive staining, bar = 100 μm. (TIFF 42085 kb) [file 12885_2018_4010_MOESM2_ESM.tif]

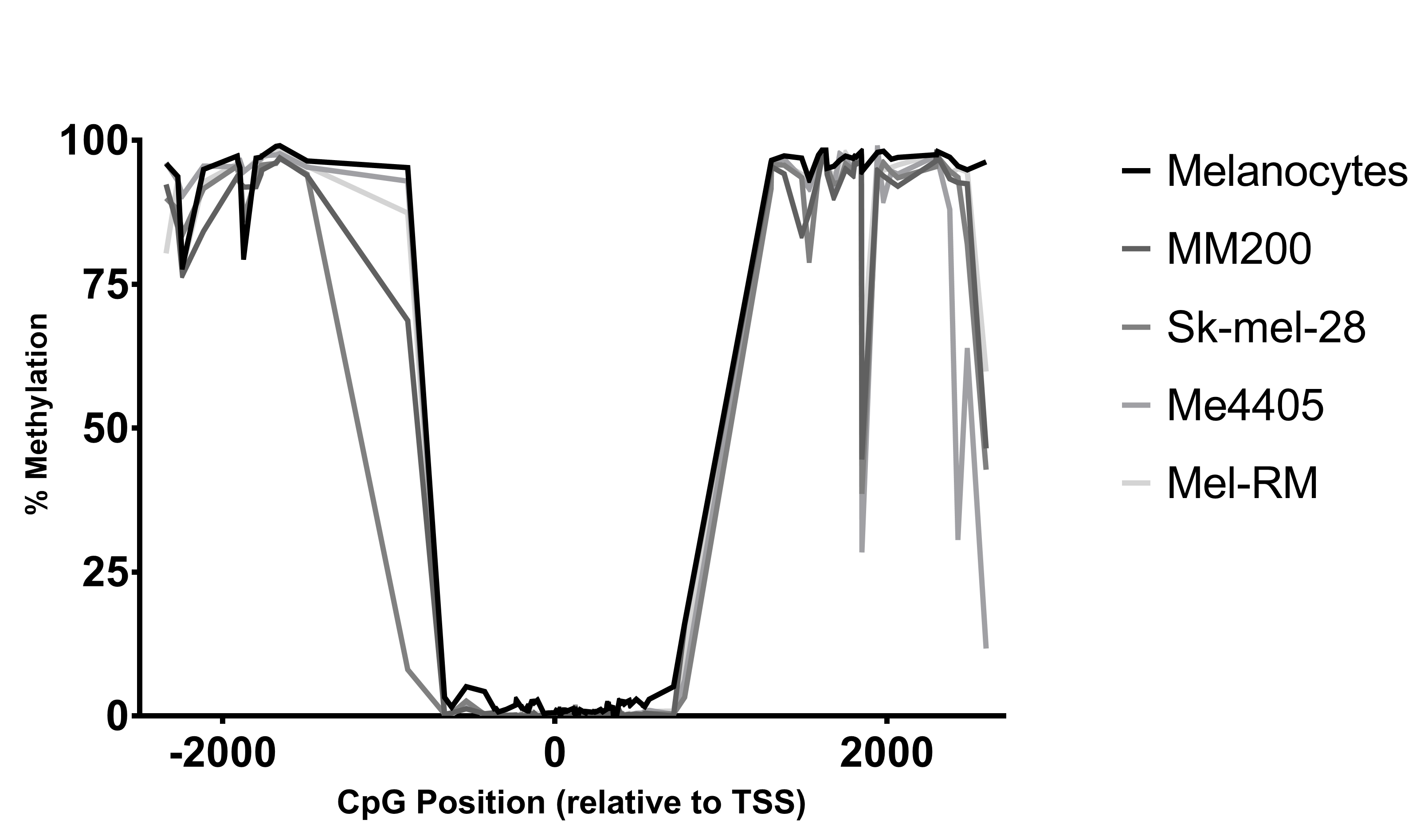

Supplement: Supplementary file 3 — DNA methylation pattern of the XPC CpG island in melanocytes and melanoma. Methylation levels in melanocytes (black) and each melanoma cell line at baseline (grey) was quantified by bisulfite sequencing. CpG position is shown relative to XPC transcription start site (TSS). Upstream (5′) shore = position − 2341 to − 423, CpG island = position − 364 to 568, Downstream (3′) shore = position 714 to 2596. (TIFF 603 kb) [file 12885_2018_4010_MOESM3_ESM.tif]
